# Supplementary material for: The Evaluation of Selected Trace Elements in Blood, Serum and Blood Cells of Type 2 Diabetes Patients with and without Renal Disorder
Source: Nutrients. 2024 Sep 4;16(17):2989. doi: 10.3390/nu16172989 (PMC11397730; doi:10.3390/nu16172989)
Supplement: Supplementary file 1 [file nutrients-16-02989-s001.zip › nutrients-3166742-supplementary.pdf]

nutrients

MDPI

# The evaluation of selected trace elements in blood, serum and blood cells of type 2 diabetes patients with and without renal disorder.

**Marcin Kosmalski [1], Rafał Frankowski [2] , Joanna Leszczyńska [3], Monika Różycka-Kosmalska [4],Tadeusz Pietras [1,5] and Iwona Majak [6,*]**

**Supplementary materials**

**Table S1.** Mean values of anthropometric measurements, selected laboratory measures, blood pressure, hematological and biochemical parameters in the study groups of patients by gender.

| Group | Control | | | T2DM and GFR > 60 | | | T2DM and GFR < 60 | | |
|---|---|---|---|---|---|---|---|---|---|
| | Overall (32) | Women (16) | Men (16) | Overall (29) | Women (17) | Men (12) | Overall (30) | Women (22) | Men (8) |
| Age [years] | 68.25±15.30 | 71.94±13.32 | 64.56±16.67 | 60.51±9.86 | 61.35±11.82 | 59.33±6.44 | 75.13±6.91 | 75.59±6.97 | 73.87±7.08 |
| BMI [kg/m$^2$] | 26.35±4.68 | 26.39±4.77 | 26.32±4.75 | 30.84±5.55 $^{a**}$ | 31.7±6.78 | 29.63±2.98 | 31.43±6.49 $^{b**}$ | 31.87±7.29 | 30.21±3.6 |
| WHR | 0.945±0.071 | 0.904±0.065 | 0.982±0.056 | 0.936±0.075 | 0.894±0.059 | 0.996±0.052 | 0.951±0.055 | 0.936±0.050 | 0.993±0.049 |
| SBP [mmHg] | 126.1±17,7 | 129.7±15.1 | 122.5±19.8 | 129.7±18.7 | 131.2±20.2 | 127.5±17.1 | 137.2±27.8 | 137.9±28.1 | 135.0±28.9 |
| DBP [mmHg] | 140.6±5.8 | 141.5±7.4 | 139.8±3.6 | 133.9±23.2 | 131.1±30.3 | 137.8±2.1 | 139.7±3.84 | 140.6±3.55 | 137.1±3.60 |
| WBC [10$^3$/µl] | 7.74±3.12 | 7.89±3.85 | 7.59±2.29 | 7.52±2.25 | 7.43±2.38 | 7.65±2.16 | 8.12±2.31 | 8.08±2.14 | 8.24±2.91 |
| RBC [10$^6$/µl] | 4.32±0.76 | 4.20±0.71 | 4.44±0.83 | 4.52±0.49 | 4.46±0.49 | 4.61±0.49 | 4.40±0.84 | 4.35±0.73 | 4.48±1.14 |
| Hb [g/dl] | 12.75±2.38 | 12.28±2.12 | 13.22±2.60 | 13.31±1.58 | 13.18±1.49 | 13.49±1.77 | 12.67±2.29 | 12.54±2.22 | 13.03±2.60 |
| HCT [%] | 38.28±6.41 | 37.2±5.43 | 39.36±7.31 | 39.97±4.04 | 39.48±4.43 | 40.66±3.47 | 38.24±6.57 | 37.80±5.90 | 39.44±8.49 |
| MCV [fl] | 89.21±9.86 | 89.75±11.81 | 88.67±7.81 | 88.56±4.62 | 88.58±3.20 | 88.54±6.28 | 87.66±4.49 | 87.26±4.11 | 88.78±5.56 |
| MCH [pg] | 31.40±11.97 | 33.4±16.4 | 29.39±4.16 | 30.10±2.26 | 29.97±1.78 | 30.29±2.88 | 29.35±2.16 | 29.32±2.17 | 29.43±2.27 |
| MCHC [g/dl] | 33.22±1.75 | 32.93±1.71 | 33,51±1.76 | 33.97±1.34 | 33.84±1.43 | 34.15±1.24 | 33.11±0.98 | 33.10±0.98 | 33.14±0.98 |
| PLT [10$^3$/µl] | 231.1±63.5 | 241.9±61.6 | 220.2±65.6 | 230.4±68.7 | 226.8±68.2 | 235.5±79.6 | 237.0±101.6 | 256.4±109.9 | 185.0±48.6 |
| Glucose [mmol/l] | 5.65±1.89 | 5.96±2.38 | 5.35±1.24 | 9.84±4.94 $^{a**}$ | 10.14±5.32 | 9.42±4.54 | 9.16±4.25 $^{b**}$ | 8.78±4.20 | 10.23±4.49 |
| HbA1c [%] | 5.75±0.46 | 5.73±0.52 | 5.76±0.41 | 8.78±2.42 $^{a**}$ | 8.83±2.50 | 8.71±2.40 | 7.98±2.39 $^{b**}$ | 7.51±1,84 | 9.24±3.30 |
| Uric acid [µmol/l] | 312.8±115.9 | 318.8±141.7 | 306.9±87.1 | 294.5±89.1 | 279.8±59.7 | 314.1±118.0 | 382.4±143.1 $^{b*,c*}$ | 371.9±145.8 | 411,3±140.6 |
| Urea [mmol/l] | 5.90±1.74 | 5.98±2.03 | 5.82±1.46 | 5.22±1.17 | 5.10±1.26 | 5.38±1.04 | 10.78±4.26 $^{b**,c**}$ | 10.38±4.37 | 11.87±4.01 |
| Creatinine [µmol/l] | 68.56±12.0 | 65.31±12.0 | 71.81±11.5 | 63.14±13.8 | 59.18±10.4 | 68.8±16.3 | 136.5±65.1 $^{b**,c**}$ | 128.5±69.3 | 158.4±48.9 |
| TCH [mmol/l] | 4.32±1.27 | 4.5±1.39 | 4.14±1.15 | 4.75±2.20 | 4.97±2.79 | 4.45±0.94 | 4.66±1.09 | 4.66±1.05 | 4.64±1.29 |
| LDL [mmol/l] | 2.67±1.21 | 2.84±1.30 | 2.50±1.14 | 2.61±0.96 | 2.46±0.89 | 2.83±1.04 | 2.89±1.08 | 2.88±1.06 | 2.92±1.20 |
| HDL [mmol/l] | 1.17±0.46 | 1.20±0.45 | 1.13±0.48 | 1.15±0.31 | 1.25±0.30 | 1.02±0.28 | 1.11±0.35 | 1.14±0.32 | 1.03±0.42 |
| TG [mmol/l] | 1.39±0.52 | 1.34±0.38 | 1.45±0.65 | 2.02±1.45 | 2.07±1.83 | 1.94±0.73 | 1.80±1.00 | 1.69±0.70 | 2.10±1.58 |
| ALT [U/l] | 25.8±15.9 $^{a*}$ | 17.4±8.3 | 33.9±17.5 | 35.7±51.5 | 24.8±23.9 | 51.6±74.0 | 22.7±13.3 | 20.3±8.5 | 29.3±21.2 |
| AST [U/l] | 29.6±28.5 $^{a*}$ | 24.2±16.4 | 35.1±36.7 | 28.2±22.2 | 23.5±17.7 | 34.8±26.8 | 26.9±17.1 | 23.5±9.7 | 36.5±28.0 |
| Bilirubin [µmol/l] | 10.92±5.03 | 9.94±3.44 | 11.89±6.20 | 10.59±6.67 | 9.46±6.07 | 12,20±7.41 | 10,57±5.57 | 9.50±4.65 | 13.51±7.07 |
| GGTP [U/l] | 29.72±24.17 | 20.19±9.89 | 39.26±30.25 | 66.90±96.63 | 36.47±28.71 | 110.0±138.3 | 39.80±34.81 | 30.59±17.12 | 65.13±56.05 |

*a: Comparison between Control and T2DM, GFR >60; b: Comparison between Control and T2DM, GFR <60; c: Comparison between T2DM, GFR>60 and T2DM, GFR <60. *P<0.05, **P<0.01.

ALT - Alanine aminotransferase, AST - Aspartate transaminase, BMI – body mass index, DBP – diastolic blood pressure, GFR – glomerular filtration rate, GGTP – gamma-glutamyl transpeptidase, Hb – hemoglobin, HbA1c – glycated hemoglobin, HCT – hematocrit, HDL – high-density lipoproteins, LDL – low-density lipoproteins, MCH – mean corpuscular hemoglobin, MCHC – mean corpuscular hemoglobin concentration, MCV – mean corpuscular value, PLT – platelets, RBC

– red blood cells, SBP – Systolic blood pressure, T2DM – type 2 diabetes mellitus, TCH – total cholesterol TG – triglycerides, WHR – waist-hip ratio. Data is expressed as mean value ± standard deviation.

**Table S2.** Significance test of the differences in the content of the studied elements in the different types of samples in groups by renal function (GFR parameter).

| | Whole blood | | Serum | | Erythrocytes | |
|---|---|---|---|---|---|---|
| **Element** | **T2DM. GFR>60** | **T2DM. GFR<60** | **T2DM. GFR>60** | **T2DM. GFR<60** | **T2DM. GFR>60** | **T2DM. GFR<60** |
| Cr | $p$>0.05 | $\boldsymbol{p}$**<0.01**** | $p$>0.05 | $\boldsymbol{p}$**<0.01**** | $p$>0.05 | $\boldsymbol{p}$**<0.01**** |
| Ni | $\boldsymbol{p}$**<0.01**** | $\boldsymbol{p}$**<0.01**** | $p$>0.05 | $p$>0.05 | $\boldsymbol{p}$**<0.01**** | $\boldsymbol{p}$**<0.01**** |
| Co | $p$>0.05 | $p$>0.05 | $p$>0.05 | $p$>0.05 | $p$>0.05 | $p$>0.05 |
| Mn | $p$>0.05 | $p$>0.05 | $p$>0.05 | $\boldsymbol{p}$**<0.05*** | $p$>0.05 | $p$>0.05 |
| Zn | $P$>0.05 | $p$>0.05 | $\boldsymbol{p}$**<0.01**** | $\boldsymbol{p}$**<0.05*** | $p$>0.05 | $p$>0.05 |

GFR – glomerular filtration rate. Co – cobalt. Cr – chromium. Mn – manganese. T2DM – type 2 diabetes mellitus. Zn – zinc. p-value assessed using the Mann–Whitney U test. *The bolded results indicate statistically significant differences at levels $p$<0.05. **The bolded results indicate highly statistically significant difference at levels $p$<0.01.

**Table S3.** Significance test of differences in elemental content by gender for each group.

| **Element** | **Gender** | **Whole blood** | | **Serum** | | **Erythrocytes** | |
|---|---|---|---|---|---|---|---|
| | | **T2DM. GFR>60** | **T2DM. GFR<60** | **T2DM. GFR>60** | **T2DM. GFR<60** | **T2DM. GFR>60** | **T2DM. GFR<60** |
| Cr | F | $p$>0.05 | $\boldsymbol{p}$**<0.05*** | $p$>0.05 | $\boldsymbol{p}$**<0.01**** | $p$>0.05 | $\boldsymbol{p}$**<0.01**** |
| | M | $p$>0.05 | $\boldsymbol{p}$**<0.01**** | $p$>0.05 | $p$>0.05 | $p$>0.05 | $\boldsymbol{p}$**<0.01**** |
| Ni | F | $\boldsymbol{p}$**<0.01**** | $\boldsymbol{p}$**<0.01**** | $p$>0.05 | $p$>0.05 | $\boldsymbol{p}$**<0.01**** | $\boldsymbol{p}$**<0.01**** |
| | M | $\boldsymbol{p}$**<0.01**** | $p$>0.05 | $\boldsymbol{p}$**<0.01**** | $\boldsymbol{p}$**<0.01**** | $p$>0.05 | $p$>0.05 |
| Co | F | $p$>0.05 | $p$>0.05 | $p$>0.05 | $p$>0.05 | $p$>0.05 | $p$>0.05 |
| | M | $p$>0.05 | $p$>0.05 | $p$>0.05 | $p$>0.05 | $p$>0.05 | $p$>0.05 |
| Mn | F | $p$>0.05 | $p$>0.05 | $p$>0.05 | $p$>0.05 | $p$>0.05 | $p$>0.05 |
| | M | $p$>0.05 | $p$>0.05 | $p$>0.05 | $\boldsymbol{p}$**<0.01**** | $p$>0.05 | $p$>0.05 |
| Zn | F | $p$>0.05 | $p$>0.05 | $\boldsymbol{p}$**<0.01**** | $\boldsymbol{p}$**<0.01**** | $p$>0.05 | $p$>0.05 |
| | M | $p$>0.05 | $p$>0.05 | $\boldsymbol{p}$**<0.01**** | $p$>0.05 | $p$>0.05 | $p$>0.05 |

GFR – glomerular filtration rate. Co – cobalt. Cr – chromium. F – females. M – males. Mn – manganese. T2DM – type 2 diabetes mellitus. Zn – zinc. *$p$-value assessed using the Mann–Whitney U test. *The bolded results indicate statistically significant differences at levels $p$<0.05. **The bolded results indicate highly statistically significant difference at levels $p$<0.01.
